# *Cpeb1* expression is post‐transcriptionally regulated by AUF1, CPEB1, and microRNAs

**DOI:** 10.1002/2211-5463.13286

**Published:** 2021-11-08

**Authors:** Souichi Oe, Shinichi Hayashi, Susumu Tanaka, Taro Koike, Yukie Hirahara, Rio Kakizaki, Sumika Sakamoto, Yasuko Noda, Hisao Yamada, Masaaki Kitada

**Affiliations:** ^1^ Department of Anatomy Kansai Medical University Hirakata Japan; ^2^ Department of Anatomy Bio‐imaging and Neuro‐cell Science Jichi Medical University Shimotsuke Japan; ^3^ Biwako Professional University of Rehabilitation Higashi‐Ohmi Japan

**Keywords:** CPEB1, AUF1, miR‐145a‐5p, let‐7b‐5p, post‐transcriptional regulation, Neuro2a cell

## Abstract

Cytoplasmic polyadenylation element binding protein 1 (CPEB1) regulates the translation of numerous mRNAs. We previously showed that AU‐rich binding factor 1 (AUF1) regulates *Cpeb1* expression through the 3’ untranslated region (3’UTR). To investigate the molecular basis of the regulatory potential of the *Cpeb1* 3’UTR, here we performed reporter analyses that examined expression levels of *Gfp* reporter mRNA containing the *Cpeb1* 3’UTR. Our findings indicate that CPEB1 represses the translation of *Cpeb1* mRNA and that *miR‐145a‐5p* and *let‐7b‐5p* are involved in the reduction in *Cpeb1* expression in the absence of AUF1. These results suggest that *Cpeb1* expression is post‐transcriptionally regulated by AUF1, CPEB1, and microRNAs.

## Graphical abstract

We have previously shown that loss of interaction between AREs in the *Cpeb1* 3’UTR and AUF1, an mRNA decay factor, increased *Cpeb1* mRNA levels but reduced the protein levels (Oe S. *et al*. 2021, Biochem Biophys Res Commun 534, 491‐497). In this study, we revealed that *Cpeb1* expression is post‐transcriptionally regulated by multiple inhibitory machineries that are regulated by AUF1, CPEB1, and microRNAs.

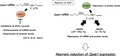

**Abbreviations**
ARE: AU‐rich element
AUF1: AU‐rich binding factor 1
CPEB1: cytoplasmic polyadenylation element binding protein 1
SG: stress granule

Cytoplasmic polyadenylation element binding protein 1 (CPEB1) is an RNA‐binding protein that interacts with cytoplasmic polyadenylation elements (CPE; consensus sequence, U_4_A_1‐2U_) on target mRNAs [1, 2]. CPEB1 plays a key role in post‐transcriptional mRNA regulation, including translational repression, mRNA localization, and formation of the mRNA‐ribonucleoprotein complex [3, 4, 5]. CPEB1‐mediated regulation of mRNA expression is involved in a variety of biological and pathological events. For example, CPEB1 regulates the translation of maternal mRNAs, including *Tex19.1* mRNA, which are essential for mouse oocyte maturation [6, 7]. In neurons, CPEB1 is one of the components of RNA granules that are mRNA‐ribonucleoprotein complexes that play critical roles in mRNA transport and activity‐dependent translation. A set of mRNAs targeted by CPEB1 are bidirectionally transported to dendrites and/or axons in a translationally dormant state and locally translated in a synaptic activation‐dependent manner [8, 9]. CPEB1‐mediated translational control contributes to synaptic plasticity and hippocampus‐dependent memory formation [10, 11]. Regarding cancer progression, CPEB1 has been shown to regulate epithelial‐to‐mesenchymal transition by modulating zona occludens 1 mRNA localization and regulating the translation of matrix metalloproteinase 9 mRNA in mammary epithelial cells [12, 13]. In hepatocellular carcinoma (HCC), *Cpeb1* expression is downregulated [14]. CPEB1 represses RNA expression levels of sirtuin 1 that upregulates the transcription of SRY‐box transcription factor 2 and Yes‐associated protein 2, which enhance tumorigenicity and chemosensitivity, respectively [14, 15]. Therefore, overexpression of CPEB1 abrogates cancer stemness and chemoresistance in HCC [14].

Considering the physiologic and pathologic significance of CPEB1, it is important to investigate the detailed regulatory mechanisms of *Cpeb1* expression. We have previously shown that the *Cpeb1* 3’UTR represses both *Cpeb1* mRNA and protein expression levels in Neuro2a cells and that loss of interaction between the AU‐rich element (ARE) in the *Cpeb1* 3’UTR and AU‐rich binding factor 1 (AUF1), an mRNA decay factor, increased *Cpeb1* mRNA levels but reduced the protein levels [16]. These findings suggest that *Cpeb1* expression is regulated by several distinct mechanisms through the *Cpeb1* 3’UTR. Interestingly, recent studies have demonstrated that a number of microRNAs (miRNAs), which function as major regulators of post‐transcriptional mRNA expression, can modulate *Cpeb1* expression by interacting with the *Cpeb1* 3’UTR in glioma [17], during mammalian spermatogenesis [18], and in neurons [19]. We thus hypothesized that *Cpeb1* expression is post‐transcriptionally regulated by multiple inhibitory mechanisms. To address this hypothesis, we focused on CPEB1‐mediated negative autoregulation, discordant role of AUF1, and miRNA‐mediated repression in *Cpeb1* expression.

## Materials and methods

### Cell culture and transfection

Mouse neuroblastoma Neuro2a cells (ATCC, Manassas, VA, USA) were cultured in Dulbecco’s modified Eagle’s medium (DMEM; Thermo Fisher, Waltham, MA, USA) supplemented with 10% fetal bovine serum (FBS; GE Healthcare, San Ramon, CA, USA). For plasmid transfection, Lipofectamine 3000 (Thermo Fisher) was used according to the manufacturer’s instructions. For small interfering RNA (siRNA) transfection, *Cpeb1* mRNA‐specific stealth RNAi siRNAs (Thermo Fisher) were designed based on the coding regions (accession number, NM_001252525). For inhibition of miRNA, mirVana miRNA inhibitors (MH11480 for *miR‐145a‐5p* and MH11050 for *let‐7b‐5p*; Thermo Fisher) were used. Transfection of siRNA and mirVana was carried out using Lipofectamine RNAiMAX (Thermo Fisher) according to the manufacturer’s instructions.

### Plasmid construction

The reporter plasmid GFP‐3’UTR was obtained as previously described [16]. Mutant reporter plasmids were synthesized using whole‐vector PCR with *Pfu* polymerase (Promega, Madison, WI, USA) and specific oligonucleotide primers. The full‐length mutant 3’UTR was digested using *BspE*I and *Bgl*II and inserted into pAcGFP‐C1. All the expression plasmids contained an SV40 PAS derived from pAcGFP‐C1 downstream of the *Cpeb1* 3’UTR. Oligonucleotide primers with mutations and their sequences are listed in Table S1.

### Quantitative PCR (qPCR)

To perform quantitative PCR (qPCR), total RNA was extracted from Neuro2a cells using Sepasol‐RNA I Super G (Nacalai Tesque, Kyoto, Japan). cDNAs were then synthesized from 1 μg total RNA by using PrimeScript RT Reagent Kit with gDNA Eraser (TaKaRa, Shiga, Japan) according to the manufacturer’s instructions. qPCR was carried out using THUNDERBIRD SYBR qPCR Mix (TOYOBO, Osaka, Japan) and the Rotor‐Gene Q real‐time PCR system (Qiagen, Hilden, Germany). Hypoxanthine guanine phosphoribosyl transferase (*Hprt*) was used as an internal control. For quantification of miRNA, RNA polyadenylation, first‐stranded cDNA synthesis, and qPCR were performed using Mir‐X miRNA qRT‐PCR TB Green Kit (TaKaRa) according to the manufacturer’s instructions. U6 small nuclear RNA was used as an internal control. Oligonucleotide sequences are listed in Table S1.

### Flow cytometry (FCM)

The GFP fluorescence intensity of Neuro2a cells transfected with reporter plasmids was measured using a FACSCanto II (Becton Dickinson, Franklin Lakes, NJ, USA) as described previously [16]. Briefly, appropriate cell populations were selected according to side scatter and forward scatter pulse areas (SSC‐A/FSC‐A). Red fluorescent protein (tagRFP; Evrogen, Moscow, Russia)‐expressing cells were distinguished from nontransfected cells by comparing FSC‐A/RFP‐A signals. GFP fluorescence intensity was measured in RFP‐expressing cells. The GFP fluorescence intensity relative to RFP fluorescence intensity was calculated to quantify the mean GFP fluorescence intensity of cells transfected with various reporter plasmids.

### Western blot analysis

Neuro2a cells were lysed in radioimmunoprecipitation assay buffer (Nacalai Tesque). SDS/polyacrylamide gel electrophoresis (PAGE) was performed using 8% and 10% SDS/polyacrylamide gel to detect CPEB1 and glyceraldehyde‐3‐phosphate dehydrogenase (GAPDH), respectively. In the case of detecting CPEB1, SDS/PAGE was performed until a prestained molecular marker protein (50 or 58 kilodalton) was fully run to the bottom of the gel to separate CPEB1 from adjacent nonspecific bands. Subsequent western blot analysis was performed using Trans‐Blot Turbo Transfer System (Bio‐Rad Laboratories, Hercules, CA, USA). Primary antibodies and horseradish peroxidase (HRP)‐conjugated secondary antibodies were used at dilutions of 1 : 500 and 1 : 1000, respectively, in 5% Blocking One (Nacalai Tesque) in Tris‐buffered saline containing 0.1% Tween 20. Proteins were visualized using Luminata Classico Western HRP Substrate (Millipore, Burlington, MA, USA) and detected using ImageQuant LAS 4000 mini (GE Healthcare). The primary antibodies used in this study were **r**abbit polyclonal antibodies against CPEB1 (ab73287, Abcam, Cambridge, UK) and GAPDH (sc‐25778, Santa Cruz Biotechnology, Santa Cruz, CA, USA). The secondary antibody was HRP‐conjugated donkey anti‐rabbit IgG antibody (Jackson ImmunoResearch, West Grove, PA, USA). The raw images of western blots are shown in Fig. S1 and Fig. S2.

### RNA immunoprecipitation assay

The RNA immunoprecipitation assay was performed as previously described [16]. Total RNAs were immunoprecipitated and extracted using an AUF1 antibody (ab61193, Abcam), CPEB1 antibody (ab73287, Abcam), HuD antibody (sc‐28299, Santa Cruz Biotechnology) and normal rabbit IgG (#2729, Cell Signaling Technology, Danvers, MA, USA). cDNA was synthesized from the precipitated RNAs by employing PrimeScript RT Reagent Kit with gDNA Eraser (TaKaRa). qPCR was performed using THUNDERBIRD SYBR qPCR Mix (TOYOBO) and Rotor‐Gene Q Real‐time PCR System (Qiagen).

### Statistical analysis

Data are presented as the mean + standard deviation of the mean of at least three experimental replicates. Statistical analyses were performed using a one‐way analysis of variance (ANOVA) followed by *the Bonferroni–Dunn test*. Significant values (*P*) are indicated in the figure legends. Error bars indicate standard deviation.

## Results

### CPEB1 represses the translation of Cpeb1 mRNA through the 3’UTR

**Fig. 1:**
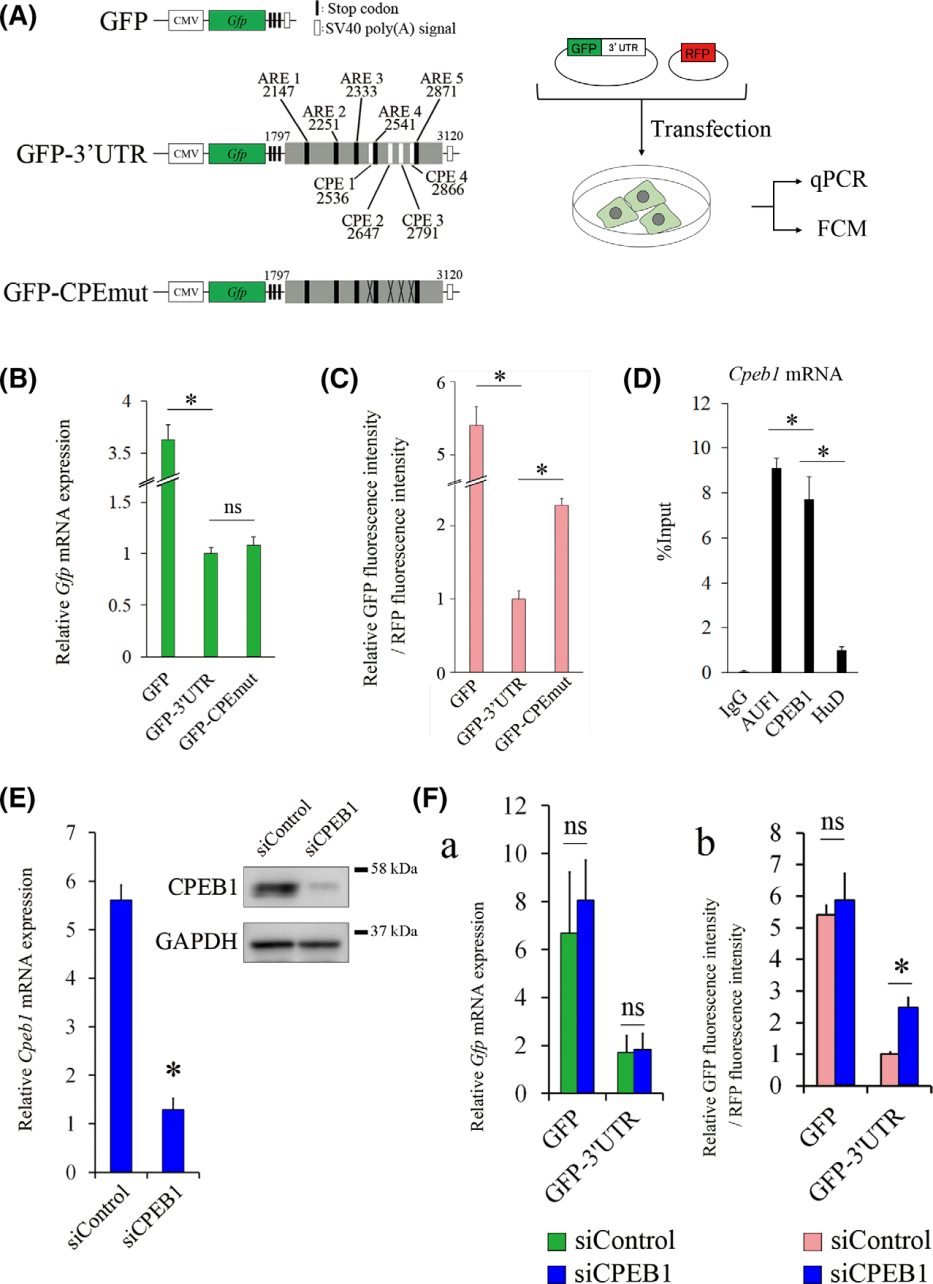
CPEB1 represses *Cpeb1* mRNA translation. (A) Schematic representation of the experimental procedure. Green fluorescent protein (GFP)‐conjugated full‐length 3’UTR sequence from nucleotide 1797 to 3120 of *Cpeb1* mRNA is shown as GFP‐3’UTR. The black and white boxes indicate ARE and CPE, respectively. The numbers indicate the position of individual ARE and CPE. A reporter plasmid that contains *Cpeb1* 3’UTR with mutated CPEs is shown as GFP‐CPEmut. Cross‐marks indicate the mutated points. Individual GFP reporter plasmid and a red fluorescent protein (RFP) plasmid were transfected to Neuro2a cells. RFP was used as a transfection control. Relative G*fp* mRNA levels and GFP fluorescence intensity in RFP‐expressing cells were measured by qPCR and flow cytometry (FCM), respectively. (B) Quantification of *Gfp* mRNA levels in GFP‐, GFP‐3’UTR‐, or GFP‐CPEmut‐transfected Neuro2a cells was performed by qPCR (*n* = 3). Relative *Gfp* mRNA expression was corrected for *Rfp* mRNA (dCt), and then, dCt of the GFP‐3’UTR is subtracted. (C). Quantification of GFP fluorescence intensity was measured by flow cytometry (*n* = 3). (D) RNA immunoprecipitation assay was performed with indicated antibodies and subsequent qPCR (n = 4). (E) *Cpeb1* mRNA and protein levels were measured by qPCR and western blotting, respectively (*n* = 4). Neuro2a cells were transfected with siControl or siCPEB1 for 24 h. GAPDH was used as a loading control. Relative *Cpeb1* mRNA expression was corrected for *Hprt* mRNA (dCt), and then, dCt of the siCPEB1 is subtracted. (F a, b) Quantification of *Gfp* mRNA levels and GFP fluorescence intensities were carried out by qPCR and flow cytometry, respectively (*n* = 4). Neuro2a cells were cotransfected with the indicated expression plasmids and siRNAs for 24 h. Relative *Gfp* mRNA expression was corrected for *Rfp* mRNA (dCt), and then, dCt of the GFP‐3’UTR is subtracted. Error bars represent standard deviation. Statistical analyses were performed using a one‐way analysis of variance (ANOVA) followed by the Bonferroni–Dunn test. * indicates *P* < 0.05, ns indicates not significant.

We previously reported that the *Cpeb1* 3’UTR represses both *Cpeb1* mRNA and protein expression levels in Neuro2a cell [16]. Furthermore, knockdown of AUF1 increased *Cpeb1* mRNA levels but reduced the protein levels. The opposite expression pattern between mRNA and protein levels suggests that other post‐transcriptional mechanisms that are distinct from ARE‐dependent mRNA decay could be involved in the reduction of protein levels CPEB1. Therefore, we first focused on CPEB1‐mediated negative autoregulation, searched for cytoplasmic polyadenylation elements (CPEs) in the *Cpeb1* 3’UTR, and identified four candidate CPEs (Fig. 1A).

To precisely analyze the roles of the 3’UTR in *Cpeb1* expression, we constructed GFP reporter plasmids: GFP‐3’UTR, which contains the 3’UTR of *Cpeb1* mRNA sequence just downstream of the stop codon to prevent aberrant translation, and GFP‐CPEmut, which contains the *Cpeb1* 3’UTR whose four CPEs were all mutated to prevent the interaction between CPEB1 and *Cpeb1* 3’UTR (Fig. 1A). We transfected the reporter plasmid and RFP‐expressing plasmid into Neuro2a cells, which is a commonly used neuronal cell line that expresses CPEB1, and estimated the expression levels of *Gfp* mRNA and protein by comparison with those of RFP by qPCR and flow cytometry, respectively (Fig. 1A). Relative *Gfp* mRNA levels in the cells transfected with GFP‐3’UTR were lower than those in cells transfected with GFP plasmid (Fig. 1B). Relative *Gfp* mRNA levels were not altered in GFP‐CPEmut‐transfected cells compared with those in cells transfected with GFP‐3’UTR (Fig. 1B). The ratio of GFP fluorescence intensity to RFP fluorescence intensity was reduced in GFP‐3’UTR‐transfected cells compared with that in GFP‐transfected cells (Fig. 1C). Interestingly, an increase in the ratio was observed in GFP‐CPEmut‐transfected cells compared with that in GFP‐3’UTR‐transfected cells (Fig. 1C). By using RNA immunoprecipitation assay, we demonstrated that endogenous *Cpeb1* mRNA pulled down by CPEB1 antibody and AUF1 antibody was significantly enriched compared with that by control IgG antibody. We also examined whether HuD, an ARE‐binding protein that enhances mRNA stability, interacts with *Cpeb1* mRNA. The amount of *Cpeb1* mRNA pulled down by HuD antibody was enriched compared with that by control IgG antibody, but lower than that by CPEB1 antibody (Fig. 1D). We next demonstrated the mRNA and protein expression levels in GFP‐3’UTR‐transfected cells that were treated with siRNA targeting the protein‐coding region of *Cpeb1* mRNA (siCPEB1). Endogenous *Cpeb1* mRNA and protein levels were significantly reduced by the addition of siCPEB1 (Fig. 1E). Although *Gfp* mRNA levels were not affected by siCPEB1 compared with siControl in GFP‐3’UTR‐transfected cells (Fig. 1Fa), the protein levels were significantly elevated in cells treated with siCPEB1 (Fig. 1Fb). These results suggest that CPEB1 interacts with CPEs in the 3’UTR and represses the translation of *Cpeb1* mRNA.

### Knockdown of both AUF1 and CPEB1 reduces *Gfp* mRNA and protein levels in the cells transfected with GFP‐3’UTR

**Fig. 2:**
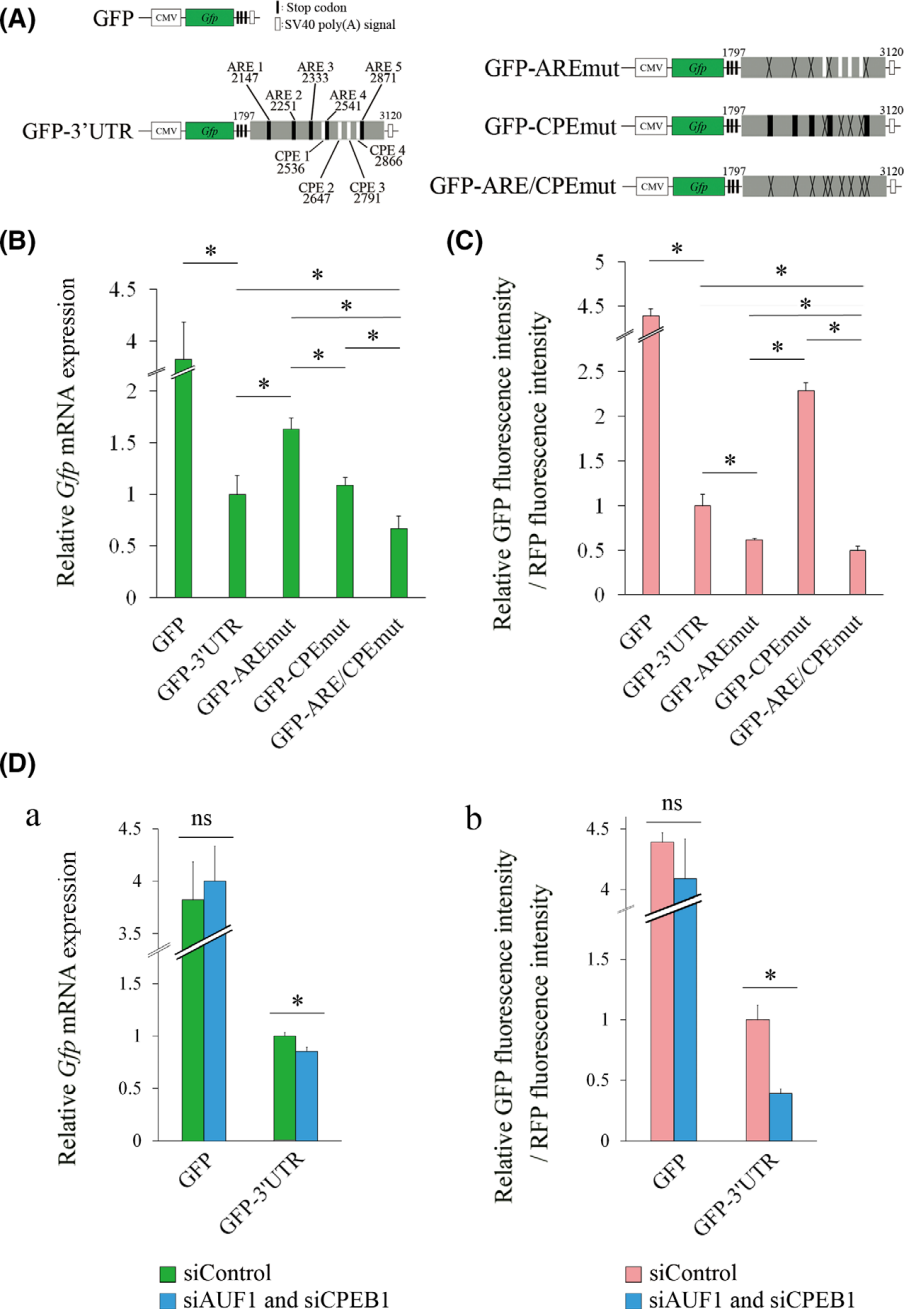
The mutation of CPE in the *Cpeb1* 3’UTR reduces *Gfp* reporter mRNA and protein levels in the absence of AREs. (A) Schematic representation of reporter plasmids. A reporter plasmid that contains *Cpeb1* 3’UTR with mutated AREs and CPEs is shown as GFP‐ARE/CPEmut. (B) Quantification of *Gfp* mRNA levels by qPCR (*n* = 4). Relative *Gfp* mRNA expression was corrected for *Rfp* mRNA (dCt), and then, dCt of the GFP‐3’UTR is subtracted. (C) GFP fluorescence intensity was measured by flow cytometry (*n* = 4). (D a, b) Quantification of *Gfp* mRNA levels and GFP fluorescence intensities were carried out by qPCR and flow cytometry, respectively (*n* = 4). Neuro2a cells were cotransfected with the indicated expression plasmids and siRNAs for 24 h. Relative *Gfp* mRNA expression was corrected for *Rfp* mRNA (dCt), and then, dCt of the GFP‐3’UTR in siControl is subtracted. Error bars represent standard deviation. Statistical analyses were performed using a one‐way analysis of variance (ANOVA) followed by the Bonferroni–Dunn test. * indicates *P* < 0.05, ns indicates not significant.

We showed that AUF1 and CPEB1 negatively regulate *Cpeb1* mRNA expression; therefore, we hypothesized that simultaneous loss of interactions between *Cpeb1* 3’UTR and both AUF1 and CPEB1 robustly increase the *Gfp* mRNA and protein levels in the cells transfected with GFP‐3’UTR. To address the hypothesis, we constructed GFP reporter plasmids, GFP‐ARE/CPEmut, in which both AREs and CPEs were mutated (Fig. 2A). After transfection of the plasmids into Neuro2a cells, we analyzed the mRNA and protein expression levels by qPCR and flow cytometry, respectively (Fig. 2B, C). In contrast to our expectation, *Gfp* mRNA and relative GFP fluorescence intensity were significantly reduced in GFP‐ARE/CPEmut compared with those in GFP‐AREmut and those in GFP‐CPEmut (Fig. 2B, C). We next examined the mRNA and protein expression levels in GFP‐3’UTR‐transfected cells that were treated with both siAUF1 and siCPEB1 (Fig. 2Da, b). *Gfp* mRNA and relative GFP fluorescence intensity were reduced in the cells transfected with both siAUF1 and siCPEB1 compared with that in the cells transfected with siControl (Fig. 2Da, b). These results suggest that simultaneous loss of interactions between *Cpeb1* 3’UTR and both AUF1 and CPEB1 represses mRNA and protein levels of CPEB1. These results are inconsistent with those in individual knockdown of AUF1 or CPEB1; therefore, we inferred that an additional alternative mechanism that negatively controls the expression of *Cpeb1* mRNA in the absence of both AUF1 and CPEB1 might exist. We thus focused on a miRNA‐mediated inhibitory mechanism in *Cpeb1* mRNA expression.

### *miR‐145a‐5p* is involved in the reduction in CPEB1 protein levels in the absence of AUF1

**Fig. 3:**
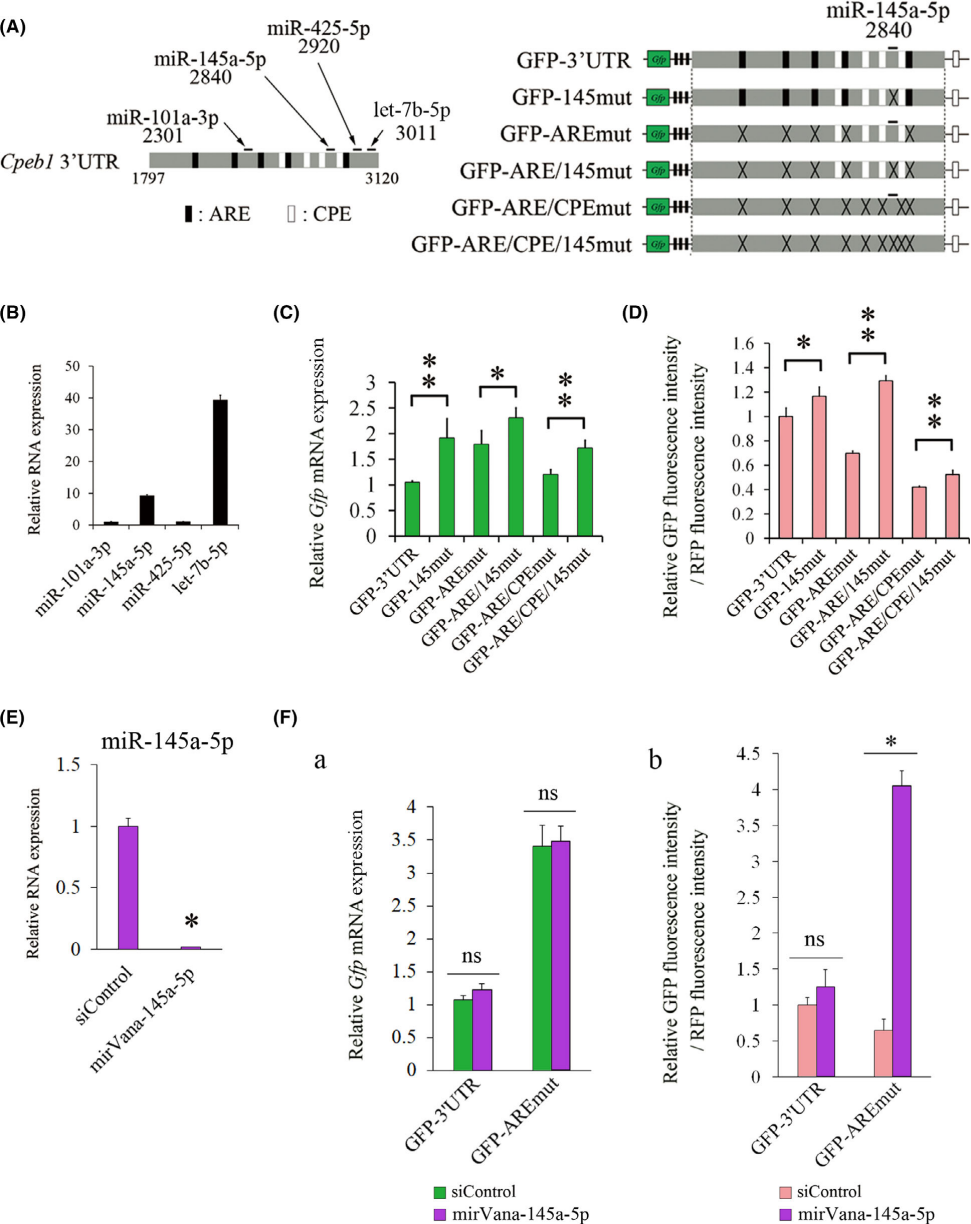
*miR‐145a‐5p* could repress *Cpeb1* mRNA and protein levels. (A) Schematic representation of ARE, CPE, and microRNA response elements (MREs) in the *Cpeb1* 3’UTR and reporter plasmids. (B) Relative expression levels of miRNAs in Neuro2a cells were analyzed by qPCR (*n* = 4). Relative miRNA expression was corrected for U6 RNA (dCt), and then, dCt of the *miR‐101a‐3p* is subtracted. (C) Quantification of *Gfp* mRNA levels by qPCR (*n* = 4). Relative *Gfp* mRNA expression was corrected for *Rfp* mRNA (dCt), and then, dCt of the GFP‐3’UTR is subtracted. (D) Quantification of GFP fluorescence intensity was measured by flow cytometry (*n* = 4). (E) *miR‐145q‐5p* expression levels were measured by qPCR (*n* = 4). Neuro2a cells were transfected with siControl or mirVana‐145a‐5p for 24 h. Relative *miR‐145a‐5p* expression was corrected for U6 RNA (dCt), and then, dCt of the siControl is subtracted. (F a, b) Quantification of *Gfp* mRNA levels and GFP fluorescence intensities were carried out by qPCR and flow cytometry, respectively (*n* = 4). Neuro2a cells were cotransfected with the indicated expression plasmids and oligonucleotides for 24 h. Relative *Gfp* mRNA expression was corrected for *Rfp* mRNA (dCt), and then, dCt of the GFP‐3’UTR in siControl is subtracted. Error bars represent standard deviation. Statistical analyses were performed using a one‐way analysis of variance (ANOVA) followed by the Bonferroni–Dunn test. * indicates *P* < 0.05, ** indicates *P* < 0.01, ns indicates not significant.

Generally, miRNAs play essential roles in both mRNA destabilization and translational repression [20]. To investigate whether miRNAs are involved in *Cpeb1* mRNA expression, we used TargetScan (http://www.targetscan.org/vert_72/) and searched for putative miRNAs that could interact with the *Cpeb1* 3’UTR. We focused on the four miRNAs that were broadly conserved among vertebrates (Fig. 3A). The miRNA expression analyses revealed that *miR‐145a‐5p* and *let‐7b‐5p* are indeed expressed in Neuro2a cells (Fig. 3B). We therefore examined whether *miR‐145a‐5p* is involved in *Cpeb1* mRNA expression. We generated reporter plasmids in which a microRNA response element (MRE) for *miR‐145a‐5p* was mutated (GFP‐145mut, GFP‐ARE/145mut, and GFP‐ARE/CPE/145mut) (Fig. 3A). After transfection of the reporter and RFP plasmids into Neuro2a cells, we examined the mRNA and fluorescence intensity by qPCR and flow cytometry, respectively (Fig. 3C, D). *Gfp* mRNA levels and relative GFP fluorescence intensity were significantly increased in GFP‐145mut, GFP‐ARE/145mut, and GFP‐ARE/CPE/145mut compared with those in the cells transfected with GFP‐3’UTR, GFP‐AREmut, and GFP‐ARE/CPEmut, respectively (Fig. 3C, D). We next examined the mRNA and protein expression levels in GFP‐3’UTR‐transfected cells that were treated with a specific inhibitory oligonucleotide for *miR‐145a‐5p*, mirVana‐145a‐5p. Endogenous *miR‐145a‐5p* level was significantly reduced by the addition of mirVana‐145a‐5p (Fig. 3E). *Gfp* mRNA was not altered but relative GFP fluorescence intensity was significantly elevated in the cells transfected with mirVana‐145a‐5p compared with that in the cells transfected with siControl (Fig. 3Fa, b). These results suggest that *miR‐145a‐5p* is involved in the reduction of protein levels in GFP‐AREmut.

### *Let‐7b‐5p* is involved in the reduction in Cpeb1 expression in the absence of AUF1

**Fig. 4:**
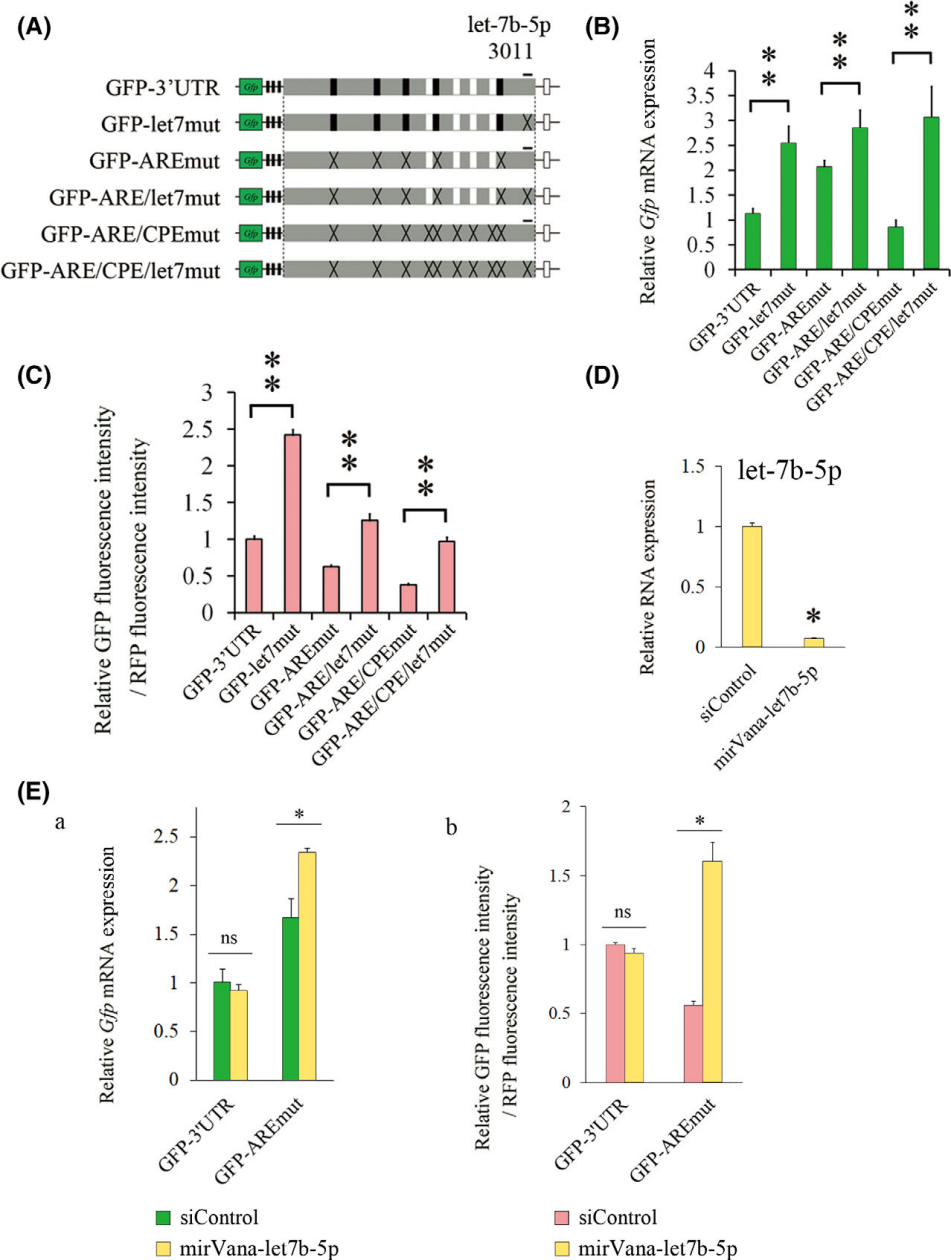
*Let‐7b‐5p* could repress *Cpeb1* mRNA and protein levels. (A) Schematic representation of reporter plasmids. (B) Quantification of *Gfp* mRNA levels by qPCR (*n* = 4). Relative *Gfp* mRNA expression was corrected for *Rfp* mRNA (dCt), and then, dCt of the GFP‐3’UTR is subtracted. (C) Quantification of GFP fluorescence intensity was measured by flow cytometry (*n* = 4). (D) *Let‐7b‐5p* expression levels were measured by qPCR (*n* = 4). Neuro2a cells were transfected with siControl or mirVana‐let‐7b‐5p for 24 h. Relative *let‐7b‐5p* expression was corrected for U6 RNA (dCt), and then, dCt of the siControl is subtracted. (E a, b) Quantification of *Gfp* mRNA levels and GFP fluorescence intensities were carried out by qPCR and flow cytometry, respectively (*n* = 4). Neuro2a cells were cotransfected with the indicated expression plasmids and oligonucleotides for 24 h. Relative *Gfp* mRNA expression was corrected for *Rfp* mRNA (dCt), and then, dCt of the GFP‐3’UTR in siControl is subtracted. Error bars represent standard deviation. Statistical analyses were performed using a one‐way analysis of variance (ANOVA) followed by the Bonferroni–Dunn test. * indicates *P* < 0.05, ** indicates *P* < 0.01, ns indicates not significant.

Next, we generated reporter plasmids in which MRE for *let‐7b‐5p* were mutated (Fig. 4A). We transfected the reporter and RFP plasmid into Neuro2a cells and examined *Gfp* mRNA levels and protein levels by qPCR and flow cytometry, respectively (Fig. 4B, C). Notably, the *Gfp* mRNA levels and relative GFP fluorescence intensity were significantly increased in GFP‐let7mut, GFP‐ARE/let7mut, and GFP‐ARE/CPE/let7mut compared with those in the cells transfected with GFP‐3’UTR, GFP‐AREmut, and GFP‐ARE/CPEmut, respectively (Fig. 4B, C). Furthermore, we analyzed the mRNA and protein expression levels in GFP‐3’UTR‐transfected cells that were treated with a specific inhibitory oligonucleotide for *let‐7b‐5p*, mirVana‐let‐7b‐5p. Endogenous *let‐7b‐5p* level was reduced by the addition of mirVana‐let‐7b‐5p (Fig. 4D). *Gfp* mRNA and relative GFP fluorescence intensity were significantly increased in the cells transfected with mirVana‐let‐7b‐5p compared with that in the cells transfected with siControl (Fig. 4Ea, b). These results suggest that *let‐7b‐5p* is involved in the reduction in protein levels in GFP‐AREmut.

### Endogenous *Cpeb1* expression is repressed by *miR‐145a‐5p* and *let‐7b‐5p*

**Fig. 5:**
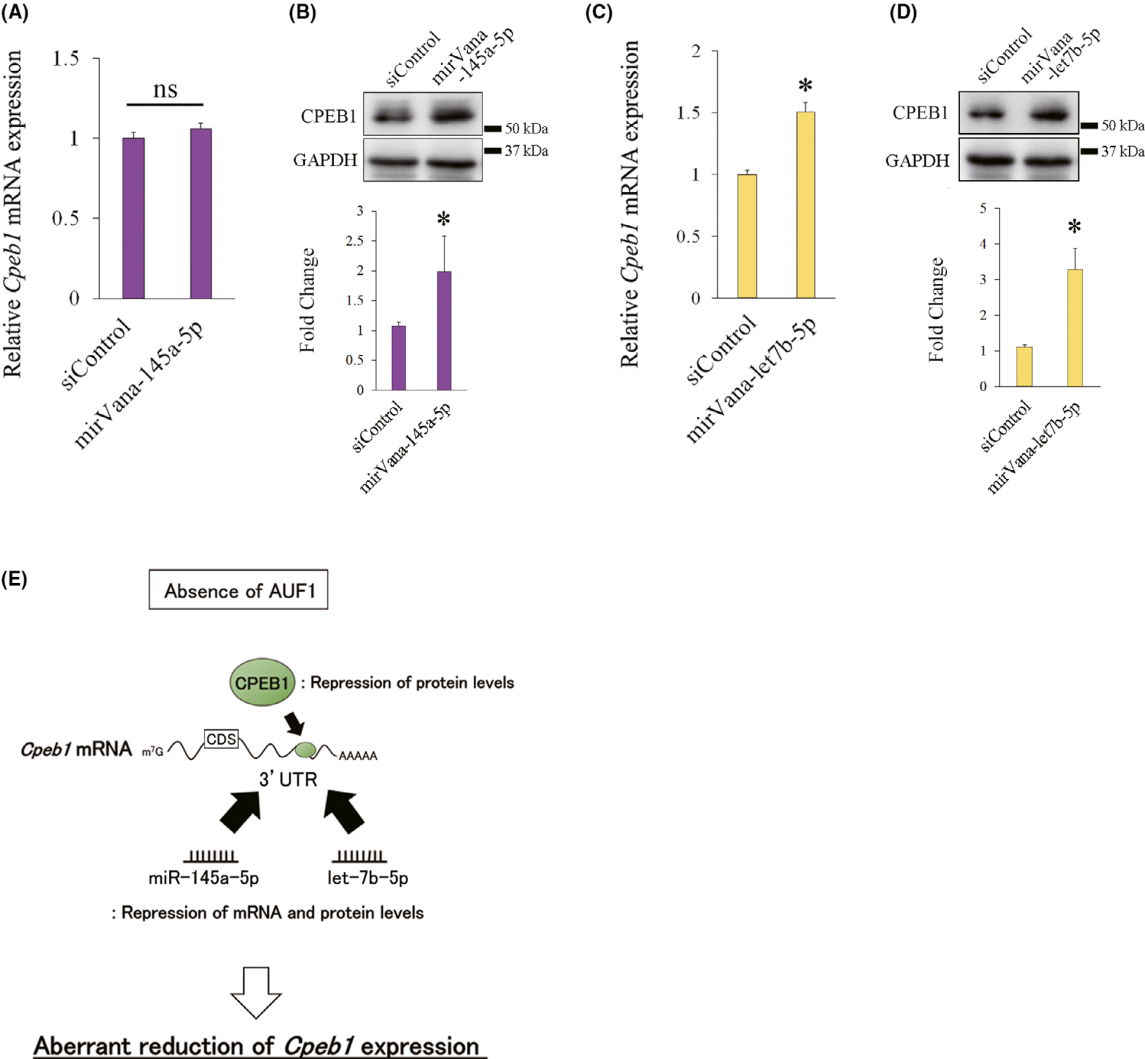
*miR‐145a‐5p* represses protein levels, and *let‐7b‐5p* represses both mRNA and protein levels in endogenous *Cpeb1* expression. (A, B) *Cpeb1* mRNA and protein levels were measured by qPCR and western blotting, respectively (*n* = 4). Neuro2a cells were transfected with siControl or mirVana‐145a‐5p for 24 h. (C, D) *Cpeb1* mRNA and protein levels were measured by qPCR and western blotting, respectively, in Neuro2a cells transfected with siControl or mirVana‐let‐7b‐5p for 24 h (*n* = 4). Relative *Cpeb1* mRNA expression was corrected for *Hprt* mRNA (dCt), and then, dCt of the siControl is subtracted. GAPDH was used as a loading control. Error bars represent standard deviation. Statistical analyses were performed using a one‐way analysis of variance (ANOVA) followed by the Bonferroni–Dunn test. * indicates *P* < 0.05, ns indicates not significant. (E) A graphical summary of the possible mechanism of *Cpeb1* expression. In the absence of AUF1, *Cpeb1* expression levels are aberrantly reduced by *miR‐145a‐5p*, and *let‐7b‐5p*.

We further examined whether endogenous *Cpeb1* mRNA and protein levels are regulated by *miR‐145a‐5p* and *let‐7b‐5p*. We transfected mirVana‐145a‐5p and mirVana‐let‐7b‐5p into Neuro2a cells and examined endogenous *Cpeb1* mRNA levels and protein levels by qPCR and western blot, respectively. *Cpeb1* mRNA levels were not changed but CPEB1 protein levels were increased in the cells transfected with mirVana‐145a‐5p compared with those in the cells transfected with siControl (Fig. 5A, B). These observations are consistent with the alternations of *Gfp* reporter expression in GFP‐3’UTR‐transfected cells that were treated with mirVana‐145a‐5p (Fig. 3Fa, b). Moreover, both *Cpeb1* mRNA and protein levels were significantly elevated in the cells transfected with mirVana‐let‐7b‐5p compared with those in the cells transfected with siControl (Fig. 5C, D). These observations are consistent with the changes in *Gfp* reporter expression in GFP‐3’UTR‐transfected cells that were treated with mirVana‐let‐7b‐5p (Fig. 4Ea, b). These results suggest that endogenous *Cpeb1* expression is indeed repressed by *miR‐145a‐5p* and *let‐7b‐5p*.

## Discussion

In this study, we aimed to reveal the mechanisms in the post‐transcriptional regulation of *Cpeb1* expression in Neuro2a cells. We have previously shown that AUF1 regulates *Cpeb1* expression through the 3’UTR, and knockdown of AUF1 upregulates *Cpeb1* mRNA expression but results in a decrease in CPEB1 protein levels [16]. To investigate the detailed mechanism, we performed a GFP reporter analysis combined with knockdown experiments.

We found that CPEB1 represses *Cpeb1* mRNA translation through the 3’UTR in Neuro2a cells. GFP protein levels of the reporter plasmids, but not mRNA levels, were elevated by mutation of CPEs in the 3’UTR (Fig. 1B, C). Furthermore, knockdown of CPEB1 enhanced GFP protein levels in GFP‐3’UTR‐transfected cells (Fig. 1Fb). RNA immunoprecipitation analysis revealed that CPEB1 indeed interacts with its own mRNA (Fig. 1D). A previous study in which the mutations of four CPEs in the *Cpeb1* 3’UTR increased translational activity at the germinal vesicle stage in mouse oocytes supports our results [21]. These findings suggest that CPEB1 negatively regulates *Cpeb1* mRNA translation through its 3’UTR.

Next, we demonstrated that mutations of both ARE and CPE in reporter plasmid significantly reduced RNA and protein levels of GFP compared with those in GFP‐3’UTR‐transfected cells (Fig. 2B, C). In addition, knockdown of both AUF1 and CPEB1 also reduced RNA and protein levels of GFP in GFP‐3’UTR‐transfected cells (Fig. 2Da, b). These findings suggest two possible molecular mechanisms regarding post‐transcriptional regulation of *Cpeb1* mRNA: (a) AUF1 and CPEB1 in combination promote stability and translation of *Cpeb1* mRNA; and (b) an alternative inhibitory mechanism is involved in the expression of *Cpeb1* mRNA especially in the absence of AUF1 and CPEB1. Considering that individual knockdown of AUF1 or CPEB1 enhanced RNA level [16] and protein level (Fig. 1Fb) of GFP in GFP‐3’UTR‐transfected cells, respectively, these *trans*‐acting factors negatively regulate *Cpeb1* mRNA expression. Therefore, we hypothesized that an alternative inhibitory mechanism exists and regulates the expression of *Cpeb1* mRNA in the absence of AUF1 and CPEB1.

Furthermore, we showed that *miR‐145a‐5p* and *let‐7b‐5p* repress *Cpeb1* expression in Neuro2a cells. In addition to several miRNAs that have previously been reported to interact with the *Cpeb1* 3’UTR [17, 18, 22, 23, 24, 25], candidate miRNAs that could interact with the *Cpeb1* 3’UTR were identified by *in silico* miRNA target database analysis by using TargetScan [26] (Fig. 3A). We then revealed that *miR‐145a‐5p* and *let‐7b‐5p* were predominantly expressed in Neuro2a cells (Fig. 3B). The *miR‐145a‐5p*‐recognition element is located between the third CPE and the fourth CPE in the *Cpeb1* 3’UTR, while an MRE that is recognized by *let‐7b‐5p* is located at the end of the *Cpeb1* 3’UTR (Fig. 3A). Mutations in these MREs augmented both the mRNA and protein levels of the reporter plasmids (Fig. 3C,D, 4B,C). Notably, endogenous *Cpeb1* mRNA and protein levels are repressed by *miR‐145a‐5p* and *let‐7b‐5p* (Fig. 5A‐D), suggesting that *miR‐145a‐5p* and *let‐7b‐5p* could repress *Cpeb1* expression in the presence of AUF1 and CPEB1. Importantly, we revealed that *Gfp* levels were elevated in the cells transfected with GFP‐ARE/CPE/145mut and GFP‐ARE/CPE/let7mut compared with those in the cells transfected with GFP‐ARE/CPEmut, and that inhibition of miRNAs augmented *Gfp* levels in GFP‐3’UTR‐transfected cells (Fig. 3Fa, b, 4Ea, b). Intriguingly, miRNA inhibitors increased protein levels of endogenous CPEB1 (Fig. 5B, D) but not altered those of GFP‐3’UTR (Fig. 3Fb, 4Eb), it is possible that GFP‐3’UTR may behave differently as compared to endogenous *Cpeb1* mRNA. Considering that GFP‐3’UTR does not possess *Cpeb1* 5’UTR and the coding region of *Cpeb1* mRNA and that several candidate MREs for *miR‐145a‐5p* or *let‐7b‐5p* indeed exist in these regions, such as 5’‐aagctgga‐3’ for *miR‐145a‐5p* and 5’‐cccacctc‐3’ for *let‐7b‐5p* in the coding region, it is plausible that interaction between *Cpeb1* mRNA and these miRNAs may be influenced by these regions. Moreover, as for the reason that *let‐7b‐5p* affected mRNA levels of GFP‐AREmut and endogenous CPEB1 (Fig. 4Ea, 5C) but *miR‐145a‐5p* did not (Fig. 3Fa, 5A), we consider two possibilities: (a) because of higher expression levels of *let‐7b‐5p* than *miR‐145a‐5p* in Neuro2a cells, *let‐7b‐5p* could exert inhibitory effects more intensively; (b) because MRE for *miR‐145a‐5p* exists immediately adjacent to CPE3 and CPE4 (Fig. 3A), it may be less effective for *miR‐145a‐5p* to interact with *Cpeb1* mRNA compared with *let‐7b‐5p*. These results suggest that *miR‐145a‐5p* and *let‐7b‐5p* are involved in the reduction in *Cpeb1* expression in the absence of AUF1 (Fig. 5E).

We demonstrated that multiple mechanisms are involved in *Cpeb1* expression. Notably, AUF1‐dependent mRNA decay and CPEB1‐mediated translational repression originally exert inhibitory effects on the post‐transcriptional regulation of mRNAs. However, our findings provide a novel insight that these inhibitory mechanisms could repress the miRNA‐mediated machinery such as the RNA‐induced silencing complex (RISC) and lead to the precise regulation of *Cpeb1* expression (Fig. 5E). How can these antagonistic actions occur? In AUF1‐dependent mRNA decay, AUF1 binds to ARE‐containing mRNAs and recruits the ribonucleases to degrade the mRNAs [27]. Considering that AUF1 predominantly localizes to the nucleus [28, 29], it is possible that AUF1 interacts with *Cpeb1* mRNA in the nucleus at an earlier stage compared with the miRNA‐mediated machinery in the cytoplasm. Although it is possible that other ARE‐binding proteins, such as HuD, are involved in *Cpeb1* expression, RNA immunoprecipitation assay in this study showed that HuD could slightly contribute in *Cpeb1* expression. In addition, since CPEB1, AUF1, and miRNAs act as widespread regulators, it is difficult to completely rule out the possibility that *Cpeb1* expression may be indirectly influenced by other regulators, such as an RNA‐binding protein HuR, whose expression levels are modulated by CPEB1, AUF1, and miRNAs [30, 31].

Interestingly, comparison of *Cpeb1* 3’UTR in mouse and humans showed that some of *cis*‐regulatory elements characterized in this study are conserved. Human *Cpeb1* mRNA seems to have two consensus CPEs that were consistent with CPE1 and CPE2, and three AREs that corresponded to ARE1, ARE2, and ARE3 in this study. Furthermore, human *Cpeb1* mRNA appears to contain MREs for *hsa‐miR‐145‐5p* and *hsa‐let‐7b‐5p* that are human orthologs of *miR‐145a‐5p* and *let‐7b‐5p*, respectively. These findings imply that human *Cpeb1* expression may be regulated by the mechanisms examined herein, although further investigations are required in future work.

A major limitation in this study is that the function of CPEB1 in miRNA‐dependent repression was not fully investigated. Generally, CPEB1‐mediated translational repression occurs at the cytoplasmic RNA‐ribonucleoprotein complex, including stress granules (SGs), which retain the ability to maintain translationally dormant state of target mRNAs [32, 33, 34]. Overexpression of CPEB1 induces the formation of SGs, suggesting that CPEB1 is a key molecule that regulates SG structure and function [5]. Intriguingly, CPEB, which is a Xenopus ortholog of CPEB1, interacts with argonaute RISC catalytic component 2 (Ago2), which is a key component of RISC formation and regulates the translation of *cyclin E1* mRNA during Xenopus oocyte maturation [35]. Furthermore, Ago2 could translocate from RISC to SGs during various kinds of cellular stresses, which significantly repress RISC‐mediated mRNA decay [36]. Although it remains unclear whether the translocation of Ago2 is regulated by CPEB1, these studies raise the possibility that CPEB1 may be related to the miRNA‐mediated inhibitory machinery.

In conclusion, our results suggest a possible mechanism by which *Cpeb1* expression is post‐transcriptionally regulated by multiple inhibitory machineries that are regulated by AUF1, CPEB1, and microRNAs.

## Conflict of interest

The named authors have no conflict of interest to the best of our knowledge, financial, or otherwise.

## Author contributions

All authors read and approved the final manuscript. MK, HY, and YN conceived and supervised the study; SO and TK designed experiments; SO, RK, and SS performed experiments; SO and YH analyzed data; SO, SH, and ST wrote the manuscript.

## Supporting information


**Fig. S1**. siCPEB1 reduced endogenous CPEB1 protein levels.

**Fig. S2**. miRNA inhibitors enhanced endogenous CPEB1 protein levels.

**Table S1**. Oligonucleotide sequences.

## Data Availability

The data that support the findings of this study are available from the corresponding author [souichi@hirakata.kmu.ac.jp] upon reasonable request.
