# Supplementary figures and images for: Cpeb1 expression is post‐transcriptionally regulated by AUF1, CPEB1, and microRNAs
Source: FEBS Open Bio. 2021 Nov 8;12(1):82–94. doi: 10.1002/2211-5463.13286 (PMC8727934; doi:10.1002/2211-5463.13286)

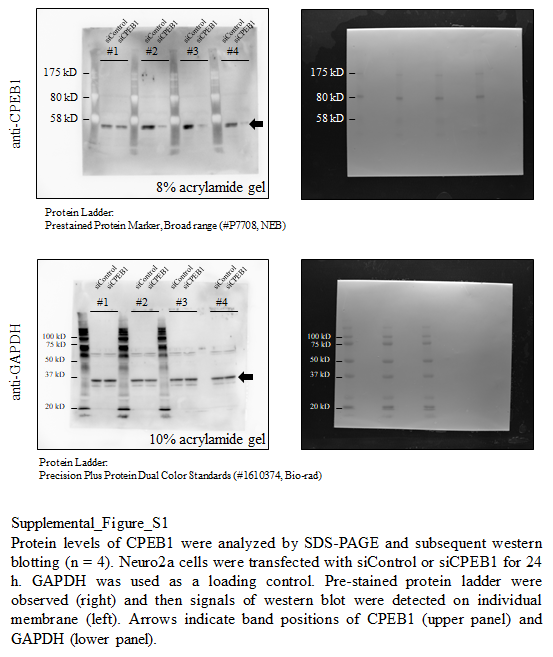

Supplement: Supplementary file 1 — Fig. S1. siCPEB1 reduced endogenous CPEB1 protein levels. [file FEB4-12-82-s003.tif]

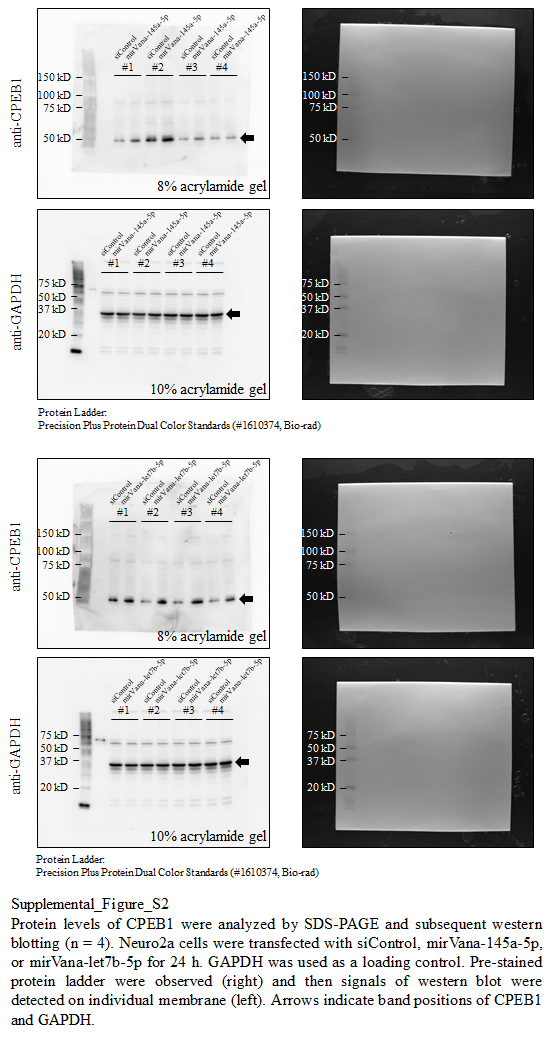

Supplement: Supplementary file 2 — Fig. S2. miRNA inhibitors enhanced endogenous CPEB1 protein levels. [file FEB4-12-82-s002.tif]
